# Tactical analysis of direct attack from the English Premier League and Spanish La Liga

**DOI:** 10.3389/fspor.2024.1473311

**Published:** 2024-10-07

**Authors:** Rodrigo Aranda-Malavés, Claudio Alberto Casal, Joaquin Gonzalez Rodenas, Andrés Tudela-Desantes, Pedro De Matías-Cid, Miquel Moltó-Llorens, Rafael Aranda

**Affiliations:** ^1^ Doctoral School, Catholic University of Valencia San Vicente Mártir, Valencia, Spain; ^2^ Department of Physical and Sports Education, Faculty of Physical Education and Sport Sciences, University of Valencia, Valencia, Spain; ^3^ Department of Physical Activity and Sport Science, Catholic University of Valencia San Vicente Mártir, Valencia, Spain; ^4^ Center for Sports Studies, Rey Juan Carlos University, Móstoles, Spain

**Keywords:** styles of play, direct attack, English Premier League, La Liga, performance analysis

## Abstract

The aim of this study was to evaluate the effect of tactical and contextual dimensions on the offensive performance, in terms of degree of penetration or creation of goal scoring opportunities (GSO), of direct attacks in the English Premier League (EPL) and Spanish La Liga. In total, 10,078 team possessions from 40 random La Liga and EPL matches were initially selected. From that, 2,384 possessions categorized as open play direct attacks were selected for analysis, including a first univariate binary regression analysis and a further construction of adjusted binary multivariate logistic regression models. Four independent tactical dimensions (“Initial zone,” “Initial opponent position,” “Initial opponent pressure,” and “Width of the possession”) and four independent contextual dimensions (“Match location,” “Match status,” “Quality of opponent,” and “Quality of the observed team”) were initially analyzed to predict penetration performance on one hand and scoring opportunity performance on the other hand. The results showed that the distribution of the type of attacks was different in La Liga and EPL (*χ*^2^ = 11.015, *p* = 0.001), but direct attacks were not different in La Liga and EPL in terms of performance. Three of the four tactical dimensions (“Initial zone,” “Initial opponent position,” and “Width of the possession”) showed association with “Penetration performance” (*p* < 0.01), while only “Width of the possession” showed association with “Scoring opportunity performance” (*p* < 0.01). Two of the four contextual dimensions (“Match status” and “Quality of observed team”) showed association with “Penetration performance” (*p* < 0.05), while none of them showed association with “Scoring opportunity performance.” In conclusion, direct attacks are frequent in EPL and La Liga but very ineffective offensively in terms of creation of goal scoring opportunities. The tactical dimensions that explain a higher effectiveness in terms of getting penetrative performance are vertical progression through the field, attacking against a defensive low-block, as well as starting the team possessions from the defensive zones instead of middle zones, while the only tactical dimension that explains higher performance in creating GSOs is vertical progression. Contextual dimensions, such as “Match status” and “Team level,” also influence the offensive penetration performance during direct attacks but not GSO performance.

## 1 Introduction

The English Premier League (EPL) and the Spanish first division (La Liga) stand as two of the most preeminent football competitions on the global stage, consistently ranking within the top three of Europe's football hierarchy over the last decade (2013–2023), as supported by Union of European Football Associations (UEFA) rankings (1). In fact, these two competitions have contributed a great amount to research studies that have provided very valuable scientific evidence about the tactical and technical characteristics and evolution of contemporary professional football in recent years (2).

In this regard, EPL seems to show a more direct style of play characterized by more frequent long balls and fast attacks than other competitions (3, 4), Nevertheless, this competition has evolved tactically in recent years (5, 6), especially driven by the highest-ranked teams, which have embraced a more possession-based style compared to their lower-ranked counterparts (7). In parallel, Spanish La Liga seems to have evolved in recent years toward a more associative and combinative game style, where the number of passes per possession and passing accuracy have increased in the last decade (8, 9).

The analysis of offensive playing styles has emerged in recent years (10–13), not only to evaluate their technical and tactical characteristics (14, 15) but also to study their offensive effectiveness (16, 17). In fact, several observational studies have identified and defined four different types of attack, such as combinative attack, fast attack, direct attack, and counterattack, based on different spatial, temporal, and technical–tactical attributes inherent in team possessions (2, 18, 19). Within this taxonomy, fast attacks and combinative attacks seem to be the most implemented by teams in EPL and Spanish La Liga, followed by direct attacks and, lastly, counterattacks (19, 20). As for offensive effectiveness, the existing evidence shows that fast attacks, particularly counterattacks, are more effective at achieving goal scoring opportunities (GSOs) and penetrative possessions than combinative attacks (2, 9, 20). Some authors suggest that it is the numerical imbalance in key spaces of the pitch that is associated with scoring goals from open play. They highlight the importance of considering not only the position in the pitch of one team player, or the position of the opponent team players, but also of considering the numerical relation between them and their numerical imbalance in each sub-space, especially in those that are close to the goal (21).

However, the number of studies that have analyzed the specific characteristics of different types of attack are still very scarce (19, 22, 23). In this regard, further research is needed to understand the tactical dimensions that are related to the offensive effectiveness of different attack types. It is particularly noteworthy that while directs attacks seem to be very ineffective offensively compared to other types of attack, they continue to constitute a substantive portion, approximately 20%–25%, of total team possessions in football (19, 20). Moreover, the prevalence of direct attacks tends to increase in away fixtures, winning scenarios, and when a team occupies a low-ranking position (24), which makes it crucial to include the effects of contextual variables in the analysis of direct attacks. Thus, the exploration of tactical and contextual variables related to the implementation and effectiveness of direct attacks could lead to a deeper understanding of this frequent type of attack. That better understanding could help prepare teams to use them in combination with other types of attacks. To be able to use a varied repertoire of attacks could lead to a better overall offensive performance (25).

Therefore, the aim of this study was to evaluate the effect of contextual and tactical dimensions on the offensive performance (degree of penetration and creation of goal scoring opportunities) of direct attacks in EPL and Spanish La Liga. We hypothesize that: (1) tactical dimensions, such as “Initial zone,” “Initial opponent position,” “Initial opponent pressure,” and “Width of the possession,” explain direct attack team performance in terms of penetration and in terms of creating goal scoring opportunities; and (2) contextual dimensions, such as “Match location,” “Match status,” “Quality of opponent,” and “Quality of the observed team,” explain direct attack team performance in terms of penetration and in terms of creating goal scoring opportunities.

## 2 Material and methods

### 2.1 Design

According to Anguera et al. (26), this observational study design was nomothetic, punctual, and multidimensional: it involved multiple units (teams) for observation, focused on a specific timeframe (one whole season, without follow-up), and assessed several dimensions. The observed behaviors took place in the teams’ usual contexts, with the observation process being direct, systematic, and non-participative. The observations were conducted using recorded matches.

### 2.2 Sample

The unit of analysis was a “team possession,” which is an open play managed by the attacking team as a direct attack. For the concept of team possession, the definition by Pollard and Reep (27) was used:

“A team possession starts when a player gains possession of the ball by any means other than from a player of the same team. The player must have enough control over the ball to be able to have a deliberate influence on its subsequent direction. The team possession may continue with a series of passes between players of the same team but ends immediately when one of the following events occurs: (a) the ball goes out of play; (b) the ball touches a player of the opposing team (e.g., by means of a tackle, an intercepted pass or a shot being saved). A momentary touch that does not significantly change the direction of the ball is excluded.”

An open play is any team possession except for the set pieces. An open play is defined as a possession that is performed in an open, adaptative way to the ever-changing position of the ball and players in the pitch. This category of possession is opposite and complementary to set pieces. A set piece is performed in a closed pre-settled way. It is always a restart of play, which is located in the opponent's half of the pitch. The team taking the set piece pre-settles a different position of its players to execute it and tries to score a goal in one or two passes (18). Otherwise, the open play could be either a restart of play or a turnover: it could start in their own or in the opponent's half of the pitch, and the teams keep the same player formation as the before and after open plays. Football is played as open play, while set pieces are spots or special situations interspersed between the open play sequences of play.

For the concept of direct attack as a specific category of open play team possession, the definition used by Aranda et al. (18) was used:

“(a) the possession starts by winning the ball in play or restarting the game, (b) the progression towards the goal is based on one long pass from the defensive players to the forward players (evaluated qualitatively), (c) the circulation of the ball takes place more in depth than in width and the intention of the team is to take the ball directly near the goal area to have opportunities of finishing by using a reduced number of passes and high tempo, (d) the opposing team has the opportunity to minimize surprise, reorganize its system, and be prepared defensively.”

Direct attack is differentially categorized and exclusive from other types of attack as combinative attack, fast attack, and counterattack (18).

For the random selection of matches, each match from the EPL and La Liga 2017–2018 season was assigned with a number from 1 to 380 in each league. An online random number generator (28) was used to select 40 random matches. The selected matches were downloaded from the Wyscout platform (29).

**Figure 1:**
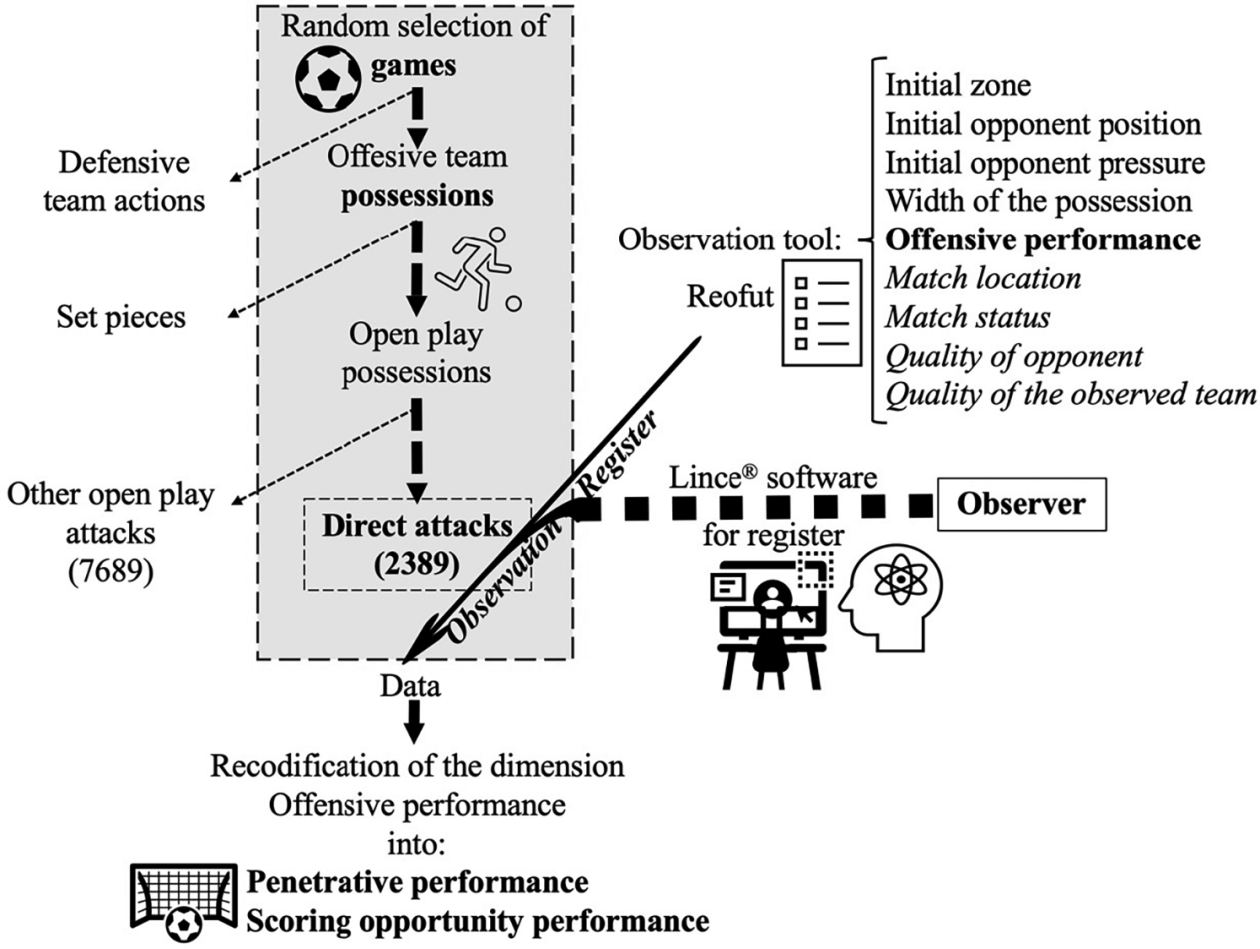
Flowchart showing the process of sample selection and the dimensions included in the observation tool, which were loaded in the Lince® software for data registration, recodification, and analysis.

In total, 10,078 team possessions from 40 random La Liga and EPL matches were initially selected. In all those possessions, all offensive team open play possessions were included, but not the set pieces. From that initial sample, only 2,394 possessions were categorized as open play direct attacks, from which 5 (0.2%) direct attack possessions could not be observed. Therefore 2,389 direct attacks were finally selected for analysis (Figure 1).

According to the Belmont Report, the use of public images for research purposes does not require informed consent or the approval of an ethical committee (30).

### 2.3 Dimensions

The REOFUT (18) is an observation tool for tactical analysis on offensive performance in football and is based on observational methodology and multidimensional analysis. This instrument describes how to analyze multiple tactical dimensions related to the start, development, and the end of teams’ possessions, as well as their association with achieving offensive performance. This instrument also considers contextual dimensions. It has been used in multiple research studies to analyze different competitions and teams. Other authors have used it to create observational instruments based on its dimensions and/or categories (3, 19, 20, 24, 31–33).

This study includes the analysis of four independent tactical dimensions, three of them related to the possession start (“Initial zone,” “Initial opponent position,” and “Initial opponent pressure”) and one related to the possession development (“Width of the possession”) (Table 1).

**Table 1:** Descriptions of independent tactical dimensions (18).

Tactical dimensions		Categories
Possession Start	Initial zone	(a) Defensive sector. (b) Pre-defensive sector. (c) Pre-offensive sector. (d) Offensive sector.
	Initial opponent position	(a) Low position: the opponent has the most backward player closer to their own goal line than the midline. (b) Medium position: the opponent has the most backward player closer to the midline than to their own goal. (c) Advanced position: the opponent has the most backward player in the opposing half.
	Initial opponent pressure	(a) Pressure: one or several opponent players press the attackers within the first 3 s of the possession [the pressing defender(s) are always located within 1.5 m from the first attackers]. (b) No pressure: There is not any player that pressures the attackers during the first 3 s of the possession.
Possession development	Width of the possession	(a) One lane: during the possession, the ball moves through one of the four longitudinal lanes. (b) Two lanes: during the possession, the ball moves through two of the four longitudinal lanes. (c) Three lanes: during the possession, the ball moves through three of the four longitudinal lanes. (d) Four lanes: during the possession, the ball moves through four of the four longitudinal lanes.

In addition, four independent contextual dimensions were analyzed: “Match location” (home, away); “Match status” (losing, drawing, winning); “Quality of the opponent”; and “Quality of the observed team” (top 5: from 1st to 5th position in the moment of the observed match; 6th–10th: from 6th position to 10th position in the moment of the observed match; 11th–15th: from 11th position to 15th position in the moment of the observed match; bottom 5: from 16th position to 20th position in the moment of the observed match).

For the evaluation of the performance, the dimension “Offensive performance” was registered. This dimension has three categories—(1) no offensive penetration; (2) offensive penetration; and (3) scoring opportunity—and analyzes the degree of penetration over the opposing defense and the creation of GSO during the direct attack.

**Figure 2:**
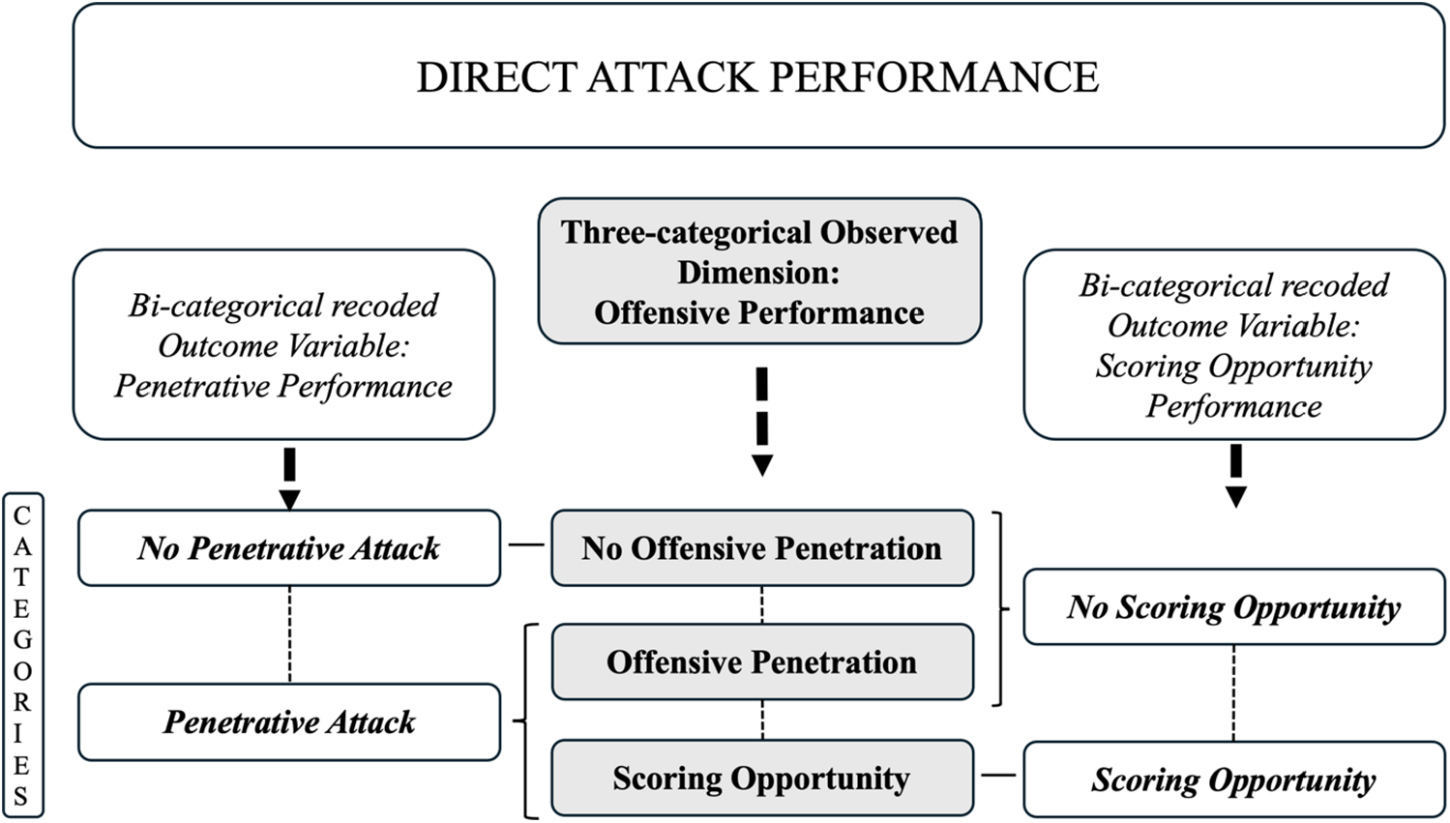
Category grouping scheme from the primary three categories of the dimension “Offensive performance” to the two new bi-categorical outcome dimensions “Penetrative performance” and “Scoring opportunity performance.”

For a more detailed analysis of the different degrees of offensive performance, the offensive performance was analyzed in two ways, by recoding its three categories into two categories: the three categories of this dimension were grouped into two categories in two different ways to obtain two bi-categorical outcome dimensions (Figure 2). The first outcome bi-categorical dimension shows the degree of penetration over the opposing defense while the second outcome bi-categorical dimension shows the degree of creation of scoring opportunities. By doing that, on one hand, it obtained a new bi-categorical outcome dimension named “Penetrative performance,” which was composed of the following two categories: the original “No offensive penetration,” renamed “No penetrative attack”; and a new category named “Penetrative attack” that comprised the other two original categories (“Offensive penetration” and “Scoring opportunity”). On the other hand, it obtained a second bi-categorical outcome dimension named “Scoring opportunity performance,” which was composed of the following two categories: the original “Scoring opportunity,” which remained with the same name; and the other two (“No offensive penetration” and “Offensive penetration”) that were grouped to create the new category “No scoring opportunity.” Recoding the three original categories of the dimension offensive performance in these two ways allows us to study two different bi-dimensional performance outcomes based on (1) offensive penetration achieved or (2) scoring opportunities created. These two new dimensions were used for the initial analysis of the association between La Liga and EPL.

### 2.4 Procedures

The study is based on the principles of observational methodology (34). For the analysis, an expert researcher with 15 years of experience in match analysis and football coaching completed a theoretical and practical training on the use of the REOFUT observation tool (18). The training consisted of a theoretical 2-h interactive review of the different dimensions and categories included in the analysis as well as their definitions. After that training, an interactive practical training session was carried out with video examples of each category and dimension. During the following week, the researcher himself analyzed several matches and selected the game situations that were doubtful, which were discussed with other experienced researchers in match analysis and coaching (two of them with UEFA-A licenses and more than 10 years of coaching experience and the third with a UEFA-Pro license and more than 20 years of coaching experience) in a further practical session, to obtain a consensus about how to categorize each specific doubtful game situation, based on previous research and functional tactical criteria. The software Lince (35) was used to observe the matches, code the dimensions and categories, and register the data.

To summarize the data collection, once the final direct attack possession was identified, the observation tool (including tactical, performance, and contextual dimensions) was loaded into the Lince® software. This software allows the user watch the game in one window while simultaneously registering and coding the direct attack according to the relevant category for each dimension in another window, using the mouse to make selections. To evaluate the reliability of data, 100 random direct attack possessions were used. Intra- and inter-observer agreement (Cohen's Kappa) was calculated for “Initial zone” (0.952 and 0.945), “Initial opponent position” (0.940 and 0.911), “Initial opponent pressure” (0.925 and 0.918), “Width of the possession” (0.931 and 0.930), and “Offensive performance” (0.941 and 0.938).

To compare the direct attack in both competitions (La Liga and EPL), this study includes an initial analysis of the association between the independent dimension “Competition” (which has two categories: “La Liga” and “EPL”), with two dependent dimensions (“Penetrative performance” and “Scoring opportunity performance”), both from direct attacks. In the same way, to compare not only the performance of the direct attack, but also the frequencies of this type of attack, the frequencies of direct attack were compared in both competitions by exploring the association between the independent dimension “Competition” and the dependent dimension “Type of attack” (which has two categories: “Direct attack” and “No direct attack”).

### 2.5 Statistical analysis

An analysis of frequencies was carried out to describe the characteristics of the sample and the occurrence of each tactical dimension according to the offensive performance.

The initial comparison of percentages of different types of attack from La Liga and EPL, as well as the association between the direct attack “Penetration performance” or direct attack “Scoring opportunity performance” with type of league was done by Pearson chi-square analysis and the effect size was calculated as the Cramer's V, qualitatively defined as small (0.10), medium (0.30), or large (0.50) (36).

As there were no differences in the direct attack performance outcomes between both competitions (La Liga and EPL), data from both competitions were analyzed together to construct the regression models that predict direct attack performance from tactical and contextual dimensions. First, a univariate analysis was carried out to determine the association of each independent tactical dimension and contextual dimension with the two performance outcomes. Second, adjusted binary logistic regression models were constructed including all significant independent dimensions (tactical or contextual) to predict the two performance-related outcomes. Those dimensions were entered into the model using the entry method. The two outcome dimensions of the regressions were: “Penetration performance” (0 = no offensive penetration, 1 = offensive penetration) and “Scoring opportunity performance” (0 = no scoring opportunity, 1 = scoring opportunity).

The level of significance was set at *p* < 0.05. All the analyses were performed using SPSS software version 20.0 (IBM Corp., Armonk, NY, USA).

## 3 Results

Table 2 shows the main characteristics of the sample. Of 5,543 EPL possessions, direct attacks accounted for 24.7% of the possessions; in La Liga, of 4,535 possessions, direct attacks accounted for 22.5% of the possessions.

**Table 2:** Frequency and percentage of the direct attacks in La Liga and EPL in the sample.

	La Liga *N* (%)	EPL *N* (%)	Both leagues *N* (%)
Direct attack	1,020 (22.5)	1,369 (24.7)	2,389 (23.7)
Other attacks	3,515 (77.5)	4,174 (75.3)	7,689 (76.3)
Total possessions	4,535 (100)	5,543 (100)	10,078 (100)

EPL, English Premier League.

The type of attack dimension showed different frequencies in La Liga and in EPL, *χ*^2^ (1) = 11.015, *p* = 0.001 with effect size V_Cramer_ = 0.046 (medium), showing that the distribution of the type of attacks is different in La Liga and EPL.

When taking only direct attack data, there was no association between the independent dimension competition (La Liga and EPL) with the dependent dimensions “Penetrative performance” and “Scoring opportunity performance,” showing that direct attacks in La Liga and EPL were not different in terms of performance, nor in the degree of penetration performance nor in the degree of scoring opportunity creation.

Table 3 shows the main characteristics of the direct attacks from La Liga and EPL, which has been the type of attack analyzed in depth. Frequencies and percentages of the two performance dimensions (“Penetrative performance” and “Scoring opportunity performance”), the four tactical dimensions, and the four independent contextual dimensions (“Match location,” “Match status,” “Quality of the observed team,” and “Quality of opponent”) are shown in Table 3 for La Liga and EPL.

**Table 3:** Descriptive characteristics of direct attacks.

		La Liga	EPL	Both leagues
Penetrative performance	No penetrative Attack	569 (55.8)	816 (59.6)	1,385 (58.0)
	Penetrative attack	451 (44.2)	553 (40.4)	1,004 (42.0)
Scoring opportunity performance	No scoring opportunity	1,005 (98.5)	1,333 (97.6)	2,341 (98.0)
	Scoring opportunity	15 (1.5)	33 (2.4)	48 (2.0)
Initial zone	Defensive	573 (56.3)	776 (56.5)	1,349 (56.4)
	Pre-defensive	337 (33.1)	422 (30.7)	759 (31.7)
	Pre-offensive	101 (9.9)	155 (11.3)	256 (10.7)
	Offensive	7 (0.7)	20 (1.5)	27 (1.1)
Opponent defensive	Despliegue	411 (40.3)	436 (31.9)	847 (35.5)
Position	Media	353 (34.6)	573 (41.9)	926 (38.8)
	Retrasada	255 (25.0)	357 (26.1)	612 (25.7)
Opponent pressure	No Pressure	516 (50.6)	751 (54.9)	1,267 (53.1)
	Pressure	504 (49.4)	616 (45.1)	1,120 (46.9)
Width	One lane	260 (25.6)	411 (30.9)	671 (28.6)
	Two lanes	507 (49.9)	638 (48.0)	1,145 (48.8)
	Three lanes	165 (16.2)	168 (12.6)	333 (14.2)
	Four lanes	84 (8.3)	113 (8.5)	197 (8.4)
Quality of team	Top 5	273 (26.8)	230 (16.7)	503 (21.0)
	6th–10th	230 (22.5)	362 (26.3)	592 (24.7)
	11th–15th	292 (28.6)	339 (24.7)	631 (26.4)
	Bottom 5	225 (22.1)	443 (32.2)	668 (27.9)
Quality of opposition	Top 5	361 (35.4)	407 (29.6)	768 (32.1)
	6th–10th	253 (24.8)	302 (22.0)	555 (23.2)
	11th–15th	223 (21.9)	336 (24.5)	559 (23.4)
	Bottom 5	183 (17.9)	329 (23.9)	512 (21.4)
Match location	Home	523 (51.3)	702 (51.0)	1,225 (51.2)
	Away	497 (48.7)	672 (48.9)	1,169 (48.9)
Match status	Drawing	557 (54.6)	657 (47.8)	1,214 (50.7)
	Wining	253 (24.8)	310 (22.6)	563 (23.5)
	Loosing	210 (20.6)	407 (29.6)	617 (25.8)

EPL, English Premier League.

For offensive tactics as predictors of the outcome “Penetrative performance,” both univariate and multivariate analysis found that pre-defensive and pre-offensive initial zones, medium and advanced position of the opponent team, and two and three lanes of width obtained lower probabilities to achieve penetrative attacks than defensive starting zone, low defensive position of the opponent, and one lane of width, respectively (Table 4).

**Table 4:** Binary logistic regression models of tactical dimensions predicting to achieve penetrative attacks vs. no penetrative attacks (reference category).

Offensive penetration vs. no offensive penetration (univariate analysis)						
		β	SE	OR	95% CI	
					Lower	Upper
Field starting zone	Defensive (Ref.)					
	Pre-defensive	−2,406	0.545	0.090***	0.031	0.262
	Pre-offensive	−2,055	0.547	0.128***	0.044	0.374
	Offensive	−0.499	0.562	0.607	0.202	1,827
	Intercept	1,749	0.542			
Opponent defensive situation	Low position (Ref.)					
	Medium position	−1,303	0.112	0.272***	0.218	0.339
	Advanced position	−0.79	0.106	0.454***	0.368	0.559
	Intercept	0.436	0.083			
Initial opponent pressure	No opponent pressure (Ref.)					
	Opponent pressure	−0.092	0.083	0.912	0.775	1,074
	Intercept	−0.279	0.057			
Width	One lane (Ref.)					
	Two lanes	−1,251	0.168	0.286***	0.206	0.398
	Three lanes	−0.748	0.158	0.473***	0.348	0.645
	Four lanes	−0.187	0.183	0.829	0.58	1,187
	Intercept	0.422	0.146			
Ref., reference category.						
Offensive penetration vs. no offensive penetration (multivariate analysis)						
		β	SE	OR	95% CI	
					Lower	Upper
Field starting zone	Defensive (Ref.)					
	Pre-defensive	−1,595	0.562	0.203**	0.067	0.61
	Pre-offensive	−1,518	0.559	0.219**	0.073	0.655
	Offensive	−0.178	0.572	0.837	0.273	2,567
	Intercept					
Opponent defensive situation	Low position (Ref.)					
	Medium position	−0.75	0.134	0.472***	0.363	0.614
	Advanced position	−0.271	0.125	0.763*	0.597	0.975
	Intercept					
Initial opponent pressure	No opponent pressure (Ref.)					
	Opponent pressure					
	Intercept					
Width	One lane (Ref.)					
	Two lanes	−0.976	0.183	0.377***	0.264	0.539
	Three lanes	−0.53	0.17	0.589**	0.422	0.822
	Four lanes	−0.197	0.195	0.821	0.56	1,204
	Intercept	2,031	0.565			

β, coefficient; SE, standard error; OR, odds ratio; CI, confidence interval for odds ratio; Ref., reference category.

* *p* < 0.05; **p < 0.01; ***p < 0.001.

For offensive tactics as predictors of the outcome “Scoring opportunity performance,” only the width dimension presented significant values in the univariate regression (Table 5), showing that two and three lanes of width obtained lower probabilities to achieve penetrative attacks than one lane of width. As only one dimension was significant, the multivariate analysis was not performed for offensive tactics and scoring opportunity performance.

**Table 5:** Binary logistic regression models of tactical dimensions predicting to achieve scoring opportunity vs. no scoring opportunity (reference category).

Scoring opportunity vs. no scoring opportunity (univariate analysis)						
		β	SE	OR	95% CI	
					Lower	Upper
Field starting zone	Defensive (Ref.)					
	Pre-defensive	−1.971	0.647	0.139	0.039	0.496
	Pre-offensive	−1.825	0.666	0.161	0.044	0.595
	Offensive	−1.492	0.722	0.225	0.055	0.927
	Intercept	−2.079	0.612			
Opponent defensive situation	Low position (Ref.)					
	Medium position	−0.447	0.338	0.64	0.33	1.241
	Advanced position	−0.892	0.372	0.41	0.198	0.851
	Intercept	−3.439	0.233			
Initial opponent pressure	No opponent pressure (Ref.)					
	Opponent pressure	0.038	0.292	1.039	0.586	1.841
	Intercept	−3.902	0.202			
Width	One lane (Ref.)					
	Two lanes	−1.489	0.482	0.226**	0.088	0.58
	Three lanes	−1.097	0.395	0.334**	0.154	0.725
	Four lanes	−0.655	0.469	0.519	0.207	1.301
	Intercept	−2.929	0.325			

β, coefficient; SE, standard error; OR, odds ratio; CI, confidence interval for odds ratio; Ref, reference category.

For contextual dimensions as predictors of the outcome “Penetrative performance,” data obtained from both univariate and multivariate analyses are shown in Table 6. Teams that were losing during the match and ranking in the bottom five of the table had lower probabilities of executing penetrative attacks compared to teams that were drawing or ranked in the top five. On the contrary, teams ranked 11th–15th and playing against opponents in the bottom five had higher probabilities of executing penetrative attacks compared to teams in the top five and playing against other top five opponents.

**Table 6:** Binary logistic regression models of contextual dimensions predicting to achieve penetrative attacks vs. no penetrative attacks (reference category).

Offensive penetration vs. no offensive penetration (univariate analysis)						
		β	SE	OR	95% CI	
					Lower	Upper
Match location	Home (Ref.)					
	Away	0.148	0.083	1.159	0.985	1.364
	Intercept	−0.398	0.000			
Match status	Drawing (Ref.)					
	Winning	−0.066	0.100	0.937	0.770	1.139
	Losing	−0.289	0.119	0.749*	0.593	0.946
	Intercept	−0.222	0.081			
Team level	Top 5 (Ref.)					
	6th–10th	0.083	0.119	1.086	0.859	1.373
	11th–15th	0.256	0.114	1.292*	1.034	1.615
	Bottom 5	−0.285	0.115	0.752***	0.601	0.942
	Intercept	−0.331	0.079			
Opponent level	Top 5 (Ref.)					
	6th–10th	−0.019	0.117	0.981	0.780	1.233
	11th−15th	0.019	0.125	1.020	0.798	1.303
	Bottom 5	0.284	0.124	1.328***	1.041	1.693
	Intercept	−0.387	0.090			
Offensive penetration vs. no offensive penetration (multivariate analysis)						
		β	SE	OR	95% CI	
					Lower	Upper
Match location	Home (Ref.)					
	Away					
	Intercept					
Match status	Drawing (Ref.)					
	Winning	−0.057	0.103	0.944	0.771	1.157
	Losing	−0.322	0.127	0.725*	0.565	0.929
	Intercept					
Team level	Top 5 (Ref.)					
	6th–10th	0.098	0.132	1.103	0.852	1.428
	11th–15th	0.244	0.124	1.276*	1.002	1.626
	Bottom 5	−0.25	0.126	0.779*	0.608	0.997
	Intercept					
Opponent level	Top 5 (Ref.)					
	6th–10th	−0.091	0.126	0.913	0.714	1.167
	11th–15th	0.055	0.136	1.056	0.809	1.38
	Bottom 5	0.103	0.136	1.109	0.850	1.447
	Intercept	−0.245	0.125			

β, coefficient; SE, standard error; OR, odds ratio; CI, confidence interval for odds ratio; Ref, reference category.

* *p* < 0.05; ***p < 0.001.

For contextual dimensions as predictors of the outcome “Scoring opportunity performance,” data obtained from the univariate analysis with the contextual dimensions as predictors were not significant (*p* > 0.05) (Table 7); therefore, a further multivariate analysis was not performed for contextual dimensions as predictors of scoring opportunity performance.

**Table 7:** Binary logistic regression models of contextual dimensions predicting to achieve scoring opportunity vs No scoring opportunity (reference category).

Scoring opportunity vs. no scoring opportunity (univariate analysis)						
		β	SE	OR	95% CI	
					Lower	Upper
Match location	Home (Ref.)					
	Away	−0.568	0.301	0.567	0.314	1.022
	Intercept	−3.635	0.185			
Match status	Drawing (Ref.)					
	Winning	−0.505	0.353	0.604	0.302	1.206
	Losing	0.098	0.37	1.103	0.534	2.277
	Intercept	−3.691	0.261			
Team level	Top 5 (Ref.)					
	6th–10th	0.426	0.371	1.531	0.74	3.167
	11th–15th	−0.451	0.447	0.637	0.265	1.529
	Bottom 5	−0.288	0.418	0.75	0.331	1.701
	Intercept	−3.839	0.27			
Opponent level	Top 5 (Ref.)					
	6th–10th	−0.267	0.398	0.766	0.351	1.67
	11th–15th	−0.641	0.48	0.527	0.206	1.349
	Bottom 5	0.129	0.392	1.137	0.527	2.454
	Intercept	−3.72	0.292			

β, coefficient; SE, standard error; OR, odds ratio; CI, confidence interval for odds ratio; Ref, reference category.

## 4 Discussion

The aim of this study was to analyze the effects of tactical dimensions and contextual variables on the offensive performance of direct attacks in La Liga and EPL football teams. The hypothesis that tactical dimensions explain direct attack team performance was confirmed for initial zone, initial opponent position, and width of the possession to influence penetration performance and for width of the possession to influence scoring opportunity performance. The hypothesis that contextual dimensions explain direct attack team performance was confirmed for match status and quality of observed team to influence penetration performance but not for any contextual dimension to influence penetration performance.

Our study found that progressing by direct attack is a frequent type of attack in both leagues (≈25%) although it is slightly more frequent in the EPL than in La Liga. It is important to note that direct attacks are just one approach to offensive play in football so that teams also employ possession-based strategies that focus on maintaining control of the ball and patiently building up attacks. Based on this fact, previous investigations have defined four types of attack: combinative; fast; counterattack; and direct [for specific definitions, see (2) and (18)]. In the last decade, it seems that there is a tendency for a higher passing frequency in European competitions over the years, indicating that football is evolving toward a more combinative and possession-oriented style of play (5, 37–39). This tendency also seems to involve a reduction in crosses and shots on goal (40), which can indicate greater difficulty in penetrating the defensive systems and thus more necessity to perform long possessions to disorganize the opposing team. In this context, direct attacks can be effective for quickly penetrating the opposing team's defense by making rapid, aggressive movements toward the goal, serving as a fast alternative to combinative or fast attacks.

However, despite the frequency of directs attacks our study, their offensive effectiveness was very low. Only 58% of these attacks managed to penetrate the defense, and only 2% resulted in GSOs. In addition, no significant differences in effectiveness were found between EPL and La Liga. In line with these findings, González-Rodenas et al. (19) observed that direct attacks were the least effective type of possession to achieve offensive penetration and goal scoring opportunities in EPL, while counterattacks and fast attacks were the most effective. Likewise, González-Rodenas et al. (20) found the same tendency in La Liga, where 38.1% and 1.7% of direct attacks resulted in penetrating the defense and creating GSOs, respectively. This low offensive effectiveness may be due to multiple factors. On one hand, direct attacks are based on a long pass from the defensive line to the forward’s line; this type of pass is normally aerial, requiring high accuracy for both the passer and the receiver. In addition, most long passes may generate an aerial or ground duel between attackers and defenders to gain possession of the ball, which very likely can cause a turnover. On the other hand, direct attacks are normally used when there is a strong defensive organization that makes it difficult to penetrate using short passes. In this context, playing direct is a quick way to put the ball near the opposing goal, but it can become predictable so that the opposing team can anticipate long passes and adjust their defensive positioning accordingly.

The key findings of our study are related to the tactical dimensions that can explain the offensive performance of direct attacks. In this regard, our results indicate that dimensions, such as playing from the defensive zone, using reduced width, and attacking an opponent in a low-block position, increased the odds of penetration compared with playing from pre-defensive or pre-offensive zones, using more width during the attack, or attacking an opponent in a medium- or high-block position. In addition, the single tactical dimension that explained the offensive effectiveness in terms of creating GSOs was the use of reduced width during the direct attack, in comparison with using two or three channels of the field. Thus, our findings suggest that effective direct attacks to penetrate defenses and achieve GSOs tend to be vertical. These attacks do not involve moving the ball from side to side across the field but focus on sending the ball directly forward through a single vertical channel. This higher effectiveness in vertical attacks may be linked to the higher speed in progression, adding an element of surprise for the opposing team. With less time to organize their defensive structure, the opposing team is more vulnerable to long passes.

Attacking against a defensive low-block also increased the odds of penetration, which suggests that achieving penetration is more likely when the defense team is not in an advanced position. The attacking team can then pass the ball from a closer position to the opposing goal, perhaps with the higher accuracy of a long pass. The lack of studies specifically analyzing direct attacks makes it impossible to compare the findings with other research. These significant results could be explained not only by factors related to the end of the possession, but particularly by those occurring at the start of the possession. To initiate a direct attack from the defensive zone might allow for long passes with less pressure, improving accuracy. In addition, playing with reduced width could be better to get key zones of the pitch faster than if during the possession the ball travels through several lanes of the pitch.

As for contextual variables, losing teams decreased the odds of penetration in comparison with drawing teams, while no significance was found regarding the creation of GSOs. In this regard, previous research has shown that losing teams had increased ball possession and the use of combinative attacks (19, 41) due to the necessity to attack to equalize the score. In this context, it is probable that the low effectiveness to penetrate the defense could be due to the urgency to send the ball close to the opposing goal by direct attacks, which may be more a desperate way to progress rather than in an organized or appropriate way to attack in that moment. In addition, highly ranked teams showed a higher probability of achieving penetration in comparison to teams ranked in the lower positions. This may be due to the higher technical accuracy of players belonging in high-ranked teams, as well as the higher capacity of the receiver of a long pass in high-ranked teams than of players in low-ranked teams. Both are more likely to achieve success in their technical–tactical actions when attacking.

To the best of our knowledge, this is the first study to analyze the incidence and offensive performance of direct attacks in professional football, specifically within the two leagues considered among the best in the world, and provide interesting practical applications. Thus, football coaches and practitioners should consider the frequency and relevancy of direct attacks in modern football by designing training sessions to improve the effectiveness of this type of attack to penetrate and create GSOs. In addition, football coaches should consider that the tactical verticality is a key dimension to penetrate and create GSOs in direct attacks in both EPL and La Liga.

The present study has some limitations. First, this study used an observational methodology to analyze and register technical and tactical events throughout the teams’ ball possessions, which may not entirely capture the high complexity of the technical–tactical performance of football. Second, the current study has been carried out with data from EPL and La Liga and the results should not be extrapolated to other leagues, other categories, or to women's football.

In conclusion, while direct attacks are frequent in EPL and La Liga, they are generally ineffective in terms of creating goal scoring opportunities. The tactical dimensions that explain higher effectiveness in terms of achieving penetrative performance include vertical progression through the field, attacking against a low-block defense, and initiating team possessions from defensive zones instead of middle zones. The only tactical dimension that explains higher performance in creating GSOs is vertical progression. Contextual variables, such as match status and team level, also influence the offense’s penetrative performance during direct attacks but do not impact GSO performance.

## Data Availability

The datasets presented in this article are not readily available because all the essential data are included in the manuscript. Requests to access the datasets should be directed to rafael.aranda@uv.es.
